# Characteristics of Unilaterally Impacted Maxillary Canines and Effect on Environmental Tissues: A CBCT Study

**DOI:** 10.3390/children10101694

**Published:** 2023-10-16

**Authors:** Ebru Kucukkaraca

**Affiliations:** Department of Orthodontics, Faculty of Dentistry, Ankara Yildirim Beyazit University, 06220 Ankara, Turkey; ebrukucukkaraca@aybu.edu.tr

**Keywords:** maxillary impacted canine, unilateral impaction, root resorption, impacted tooth morphology

## Abstract

Background: The prognosis of orthodontic treatment for a patient with impacted canine teeth can be affected by many factors and understanding some of the characteristics of impacted teeth can increase the effectiveness and reduce the duration of treatment. This study aims to explore the effects of positions and dentoalveolar morphological characteristics of impacted maxillary canines on the prognosis of orthodontic treatment. Methods: In this study, forty-six adolescent individuals who applied for treatment of impacted teeth were divided into three groups: CC (no impacted maxillary canine group), BC (unilateral buccally positioned impacted maxillary canine group), and PC (unilateral palatally positioned impacted maxillary canine group). The impacted canine and adjacent teeth were evaluated in terms of position, angulation, inclination, mesiodistal and buccolingual widths, root length, dilaceration angle, and root resorption. Results: The mean values of U3 Ang (angulation of the maxillary impacted canine) and U3/U2 angles (maxillary impacted canine and lateral incisor angle) were statistically significantly higher in the BC and PC groups (*p* < 0.001). The mean values of U2 RL (lateral incisor root length) and U3 RD (canine root dilaceration angle) were statistically significantly lower in the BC and PC groups. Conclusions: The results of this study suggest that shorter root lengths and increased angulation values may lead to the ectopic eruption of canines. The higher canine angulations in the PC group increase the degree of root resorption.

## 1. Introduction

Maxillary canines show eruption abnormalities characterized by frequent ectopic eruptions or impactions because they are between teeth that erupt later than other teeth in the dental arch and follow a long and difficult eruption pathway higher than the other teeth during the eruption. Knowing the basic concepts of development and eruption will help to understand the cause of malposition and choose the appropriate treatment [1,2].

Maxillary canines are the most commonly impacted teeth in the dental arch, following the third mandibular molars. Dachi and Howell conducted a study on full-mount radiographs from patients over the age of 20 and found a 0.92 incidence of impacted maxillary canines. They stated that these cases were mostly unilateral and saw twice as many women as men. The incidence of mandibular canines was approximately 0.09 [3].

Two major theories (genetic theory and guidance theory) explain the cause of the ectopic persistence of permanent maxillary canines. According to the guidance theory, the roots of the maxillary lateral incisor guide the eruption of the maxillary permanent canine in the dental arch in the proper direction. The canine is guided by the roots of the previously erupted permanent laterals to its normal position. In cases such as hypoplasia or lateral incisor aplasia, the canine crown cannot continue in its proper place and becomes palatal dysplasia. According to the genetic theory, ectopic eruption, or impaction of the canine in the palatal direction, is due to genetic reasons. In ectopic eruptions or impaction due to genetic reasons, the same problems can be seen in other family members [1,4,5,6].

The mesiodistal localization and angulation of the canines are important regarding their eruption potential. Ericson and Kurol reported that permanent canine crowns positioned mesially are less likely to erupt after the extraction of deciduous canines. The proportion of impacted canines in the buccal/labial direction is less than that of impacted canines in the palatal direction. Impacted maxillary canines are 85% palatal and 15% labial/buccal [7]. During their normal development, canines tend to erupt in the palatal direction between the ages of 5 and 9, while they move more in the buccal direction between the ages of 10 and 12 [8]. Space and crowding in the maxillary dental arch are often the main causes of buccally located canines, while excess space and lateral deficiency cause palatal-located canines [9]. Palatal-positioned ectopic canines are more common in cases of excess space in the maxillary dental arch, lateral deficiency or shape anomaly, and Angle Class II Division 2 malocclusion [10]. In his study, Becker found that 5.5 percent of palatally displaced canines did not have maxillary lateral incisors. This was 2.4 times higher than in the general population [11].

Cone beam computerized tomography (CBCT) is a more accurate method, in comparison to traditional radiographs, for defining the position of the impacted canine in three dimensions and analyzing whether there is root resorption [9]. In addition, CBCTs are very good at analyzing follicle size, the buccal and palatal position of the tooth, inclination of the long axis of the tooth, the amount of bone in the tooth, the condition of adjacent teeth, root resorption, and dental development mechanisms in dental follicle research [12].

Treatment of impacted canines often involves a complex multidisciplinary approach that requires surgery, periodontology, restorative treatment, and orthodontic applications. A clinical and radiological evaluation must be assessed on a patient with an impacted canine to fully evaluate the existing malocclusion and the prognosis of orthodontic treatment for the location of the impacted tooth. Age, cooperation, oral hygiene, skeletal variation, spacing or crowding in the dental arch, and the vertical, anteroposterior, and transverse positions of the canine crown or root influence the prognosis. The prognosis is poor if the canine is placed close to the midline and at an angle of more than 45° to the midline. Furthermore, for successful treatment, the root should not have ankylosis or dilaceration and should be carefully examined to determine any resorption of the incisor roots. The more the tooth is moved, the poorer the prognosis. A good buccal overjet-overbite relationship and a correct root position ensure a stable outcome [13,14,15].

Furthermore, if the canine is above the occlusal plane, the prognosis of treatment is poor. McShery defined the “vertical rule of thirds” to determine the vertical level of impacted canines. He stated that if the apex of the canine tubercle is at the level of the adjacent incisor’s enamel-cementum junction, the prognosis of treatment is good. The prognosis is moderate at half the length of the incisor root and poor at the apical third [16]. If the canine apex is 14 mm above the occlusal plane, treatment takes an average of 24 months; if it is more than 14 mm, treatment can take up to 31 months [17].

Pitt et al. investigated the factors affecting the difficulty of treatment. They found that the ectopic canine’s horizontal position, the age, the BL position, or the vertical height of the ectopic tooth are among the factors affecting orthodontic treatment [18].

Root resorption was observed in 38 percent of the laterals and 9 percent of the central incisors due to ectopically located canines, but only 3 percent of normal erupting canines had root resorption in the lateral incisors. Once resorption occurs, it progresses quite rapidly. Therefore, the diagnosis and treatment decisions should be made very quickly and resorption occurs more frequently in palatal canines [19]. When impacted canines are not treated, they can cause external root resorption, internal resorption, cyst formation, migration of adjacent teeth, crown resorption of the ectopic tooth, and labial or lingual malposition of the ectopic tooth [14,20].

Knowing some of the characteristic features of impacted teeth may increase the effectiveness of treatment and shorten the duration. This study aims to explore the effects of vertical, horizontal, and transversal positions and the dentoalveolar morphological characteristics of impacted maxillary canines on the prognosis of orthodontic treatment, and additionally detect the existence of crown root anomalies or resorption that may occur in impacted teeth or adjacent teeth. The current study’s null hypothesis is that no statistically significant differences in morphological features exist between the unilaterally impacted maxillary canines and the nonimpacted canines.

## 2. Materials and Methods

### 2.1. Ethics Approval

The project was approved by the ethics committee of Ankara Yildirim Beyazit University, Ankara, Turkey (ref: 08.12.2022/19-1272).

### 2.2. Calculation of the Sample Size and Participants

This study was planned as a cross-sectional study. CBCT images of 46 patients with impacted canines who applied to the Department of Orthodontics, Faculty of Dentistry of Ankara Yildirim Beyazit University for the treatment of impacted teeth were used. All CBCT images were taken with a Promax 3D Mid machine (Planmeca, Helsinki, Finland). The optimum exposure parameters 94 kVp, 14 mA, and 27 s were used for exposure. A single orthodontist evaluated the CBCT images in 3 sections (sagittal, axial, and coronal). All angular and dimensional measurements were made using the Planmeca Romexis 3D Imaging Program (Planmeca Romexis 3D Imaging Software, Helsinki, Finland).

The G*Power software was used to calculate the sample size (version 3.1.9.6; Franz Faul Universitat, Kiel, Germany). The power analysis was calculated with reference to the angle of the impacted canine to the midline, as evaluated in the study by Al-Tawachi et al. [21]. According to the power analysis, the total sample size was determined to be 27, with a desired power (1-b) of 0.95 at the conventional a level (0.05) and an effect size of 0.84.

### 2.3. Inclusion and Exclusion Criteria

Inclusion criteria:

- adolescents aged 10–16;
- unilateral impaction of permanent maxillary canines;
- presence and eruption of the maxillary lateral incisors;
- CBCT images with good image quality.

Exclusion criteria:

- no systemic or dentofacial deformities;
- without syndromic or cleft palate;
- without orthodontic treatment;
- no contributing history of trauma;
- no multiple missing teeth;
- no presence of cysts or other pathology.

### 2.4. Study Design and Definitions of the Variables Used in the Study

**Figure 1:**
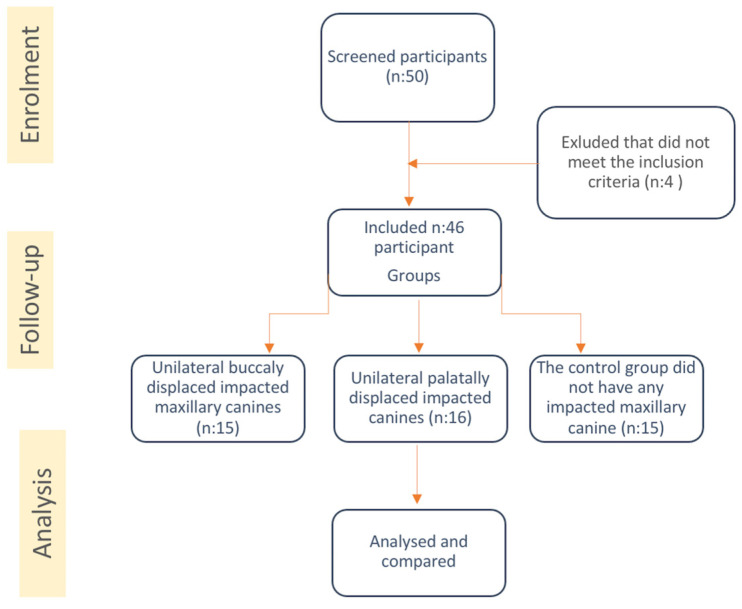
Flow chart showing the methodology of the study.

The samples were divided into three groups based on the location of the impacted canine:

- no impacted maxillary canine group (CC, control group);
- unilateral buccally positioned maxillary impacted canine group (BC);
- unilateral palatally positioned maxillary impacted canine group (PC) (Figure 1).

The demographic characteristics of the participants (age and gender) were identified. The following canine variables were measured: side of impaction (left or right); location (vertical or horizontal); rotation (mesiobuccal, distobuccal, mesiopalatal, or distopalatal); level of contact relationship with the adjacent tooth (crown, cervical, middle, or apical); impacted canines with the sagittal view (sectors 1–5); root resorption levels and number of adjacent teeth (grade 0–3).

**Figure 2:**
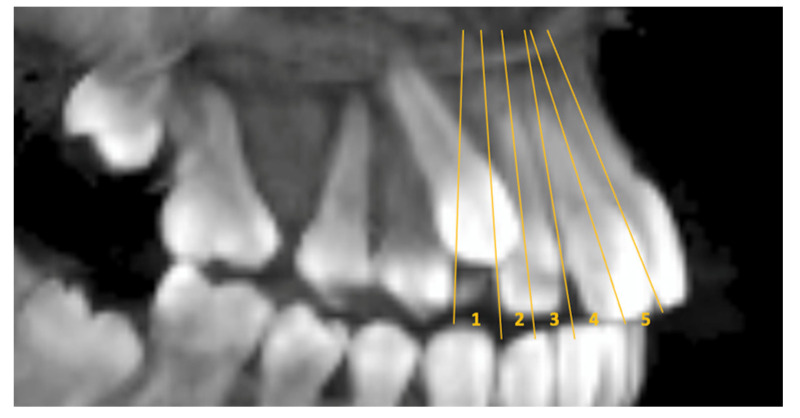
Sagittal view of the reference planes indicating the sectors.

Sector: some reference planes are used to evaluate the sagittal position of the canine and the amount of overlap (sectors) with other teeth. These are divided into 5 categories: 1 = in normal position; 2 = to the plane passing through the mid-long axis of the lateral incisor; 3 = to the plane tangent to the mesial part of the lateral incisor; 4 = to the plane passing through the mid-long axis of the central incisor; 5 = to the plane tangent to the mesial part of the central incisor [22] (Figure 2).

Resorption: the resorption in the adjacent teeth was evaluated using axial CBCT images. The Ericson–Kurol classification was used to grade the degree of resorption. Grade 0 (no resorption): intact root surface except for cement loss; grade 1 (mild resorption): mild resorption of the pulp up to half the thickness of the dentin; grade 2 (moderate resorption): half or more of the pulp is covered with pulp dentin; grade 3 (severe resorption): the pulp is exposed [23].

**Figure 3:**
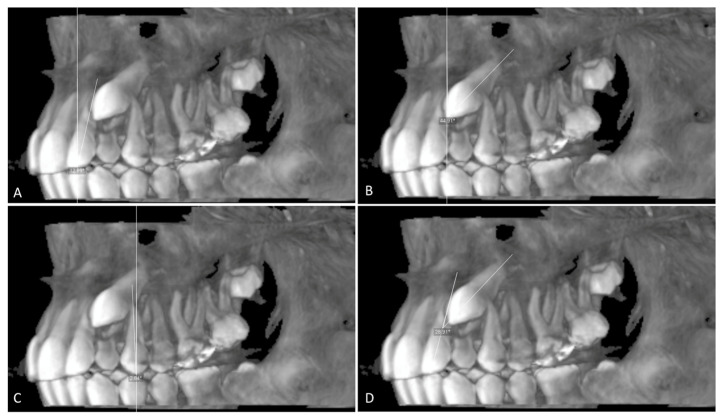
(**A**–**C**) Angulation of lateral incisor (U2 Ang), canine (U3 Ang), and adjacent first premolar (U4 Ang); the angle between a vertical line and a long axis line of the lateral incisor, canine and first premolar (**D**) (U3/U2); the angle between the long axes of the lateral incisor and canine.

The angulation of the maxillary impacted canine (U3 Ang), the lateral incisor (U2 Ang), and the first premolar (U4 Ang) is the angle between the vertical line and long axis line of the lateral incisor, the canine, and the first premolar. The angle between the impacted canine and lateral incisor (U3/U2) is the angle between the long axis of the impacted canine and the long axis of the adjacent lateral incisor (Figure 3).

**Figure 4:**
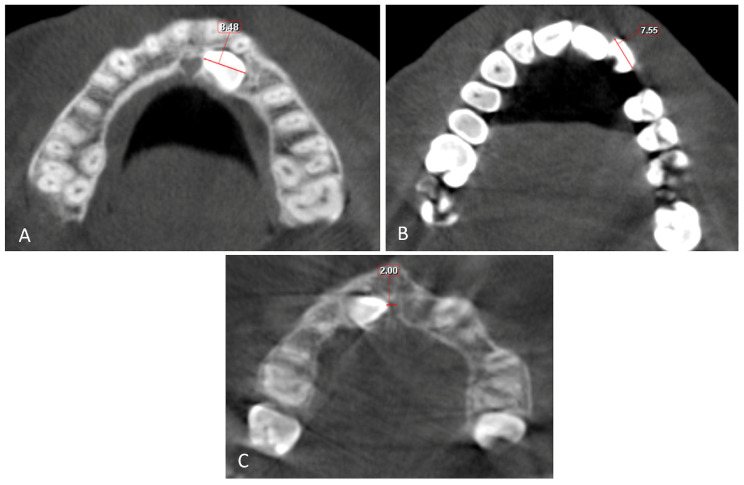
(**A**) Mesiodistal width of the canine crown (U3 MD). (**B**) Mesiodistal width of the lateral crown (U2 MD); the distance between widest points of the crown on the axial section. (**C**) Horizontal distance to midline from canine crown (U3 HD); distance from cusp tip of canine crown to the midline on the axial section.

**Figure 5:**
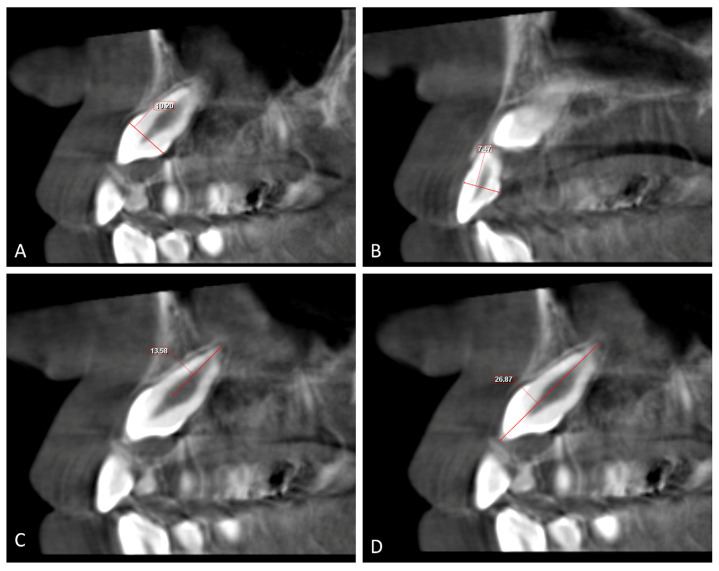
(**A**) Buccolingual width of the canine crown (U3 BL). (**B**) Buccolingual width of lateral crown (U2 BL); the distance between widest points of the crown on the sagittal section. (**C**) Length of the root canine (U3 RL) and length of the root lateral incisor (U2 RL); the length from the cementoenamel junction (CEJ) to the root apex. (**D**) Total length of the canine and lateral incisor (U3 L, U2 L); the length from the incisal tip to the root apex on the sagittal section.

The buccolingual (BL) and mesiodistal (MD) widths of the crowns of the lateral incisor (U2) and canine (U3) on the sagittal and coronal sections were measured from the widest point of the crown perpendicular to the long axis (Figure 4 and Figure 5).

**Figure 6:**
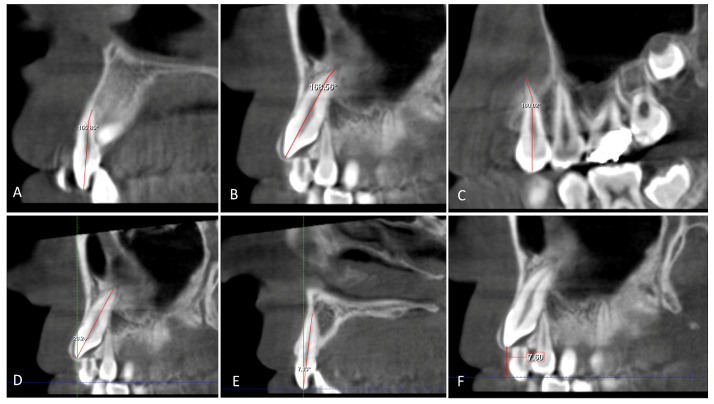
(**A**–**C**) The angle root dilaceration of canine (U3 RD), lateral incisor (U2 RD), and first premolar (U4 RD); the angle between a line to long axis of tooth and a line of the root apex. (**D**,**E**) Inclination of canine (U3 Inc) and lateral incisor (U2 Inc); the angle between a line 90° and a line to the long axis of the canine and lateral incisor. (**F**) Vertical distance to occlusal plane from canine crown (U3 VD); distance from cusp tip of canine crown to the occlusal plane on the sagittal section.

Canine root length (U3 RL) and lateral root length (U2 RL) were measured from the crown-root junction to the root apex, and the total length (U3 L, U2 L) of the lateral incisor and canine was measured from the incisal crown tip to the root apex on the sagittal view (Figure 5). The horizontal distance of the maxillary impacted canine (U3 HD) from the tip of the canine cusp to the midline was measured in the axial section. The vertical distance of the maxillary impacted canine (U3 VD) from the tip of the canine cusp to the occlusion plane was measured in the sagittal section (Figure 4, Figure 5 and Figure 6).

The inclination of the maxillary impacted canine (U3 Inc) and lateral incisor (U2 Inc) is determined by measuring the angle between the plane passing through the long axis of the lateral incisor and canine and a vertical plane in the sagittal section. (Figure 6). The root dilaceration angle of the canine (U3 RD), lateral incisor (U2 RD), and 1st premolar (U4 RD) is the angle between a line through the long axis of the tooth and a line of the root apex (Figure 6).

### 2.5. Statistical Analysis

The data were analyzed using the SPSS program (Version 29; Inc., Chicago, IL, USA). In the study, the analysis of the normal distribution of the measurements was performed using the Shapiro–Wilk test. Mean ± SD, which are descriptive statistical data for the variables, are given in Table 1 The demographic characteristics of the participants were analyzed using the chi-square test or Fisher’s exact test when the number of subjects in any group was <5 [24]. The percentages of location, rotation, sectors, contact point, and resorption of impacted canines in the groups were analyzed using the chi-square test (Fisher–Freeman–Halton exact test). Differences in linear and angular measurements between the groups were evaluated using a one-way ANOVA test for values with a normal distribution and the Kruskal–Wallis test for nonnormally distributed values. Tukey (for the homogeneously distributed variables) and Tamhane (for the non-homogeneously distributed variables) post hoc tests were used to determine statistically different values between groups. A *p* < 0.05 was considered significant.

## 3. Results

This study consisted of 22 males (47.8%) and 24 females (52.2%). The mean age of the included subjects was 14.58 ± 2.29 years. The side of impaction showed that 23 (50%) of the impacted canines were located on the left and 23 (50%) on the right side (Table 1). The demographic characteristics of the participants were analyzed using the chi-square test (Fisher’s exact test) (Table 1).

In general, forty-two (91.3%) of the forty-six impacted canines were vertical, while four (8.7%) were horizontal. A statistically significant positive association (c^2^ = 6.02, *p* < 0.05) was found between the groups. The impacted canine rotation was found to have the highest percentage of mesiopalatal rotation (54.3%). Distobuccal and distopalatal rotations were not observed. Distributions of contact relations, location, rotations, sectors, and severity of resorption are analyzed by the chi-square test (Fisher–Freeman–Halton exact test) and are presented in Table 2. A statistically significant positive association (c^2^ = 48.08, *p* < 0.01) was found between the groups (Table 2).

The highest percentage was observed in Sector 1 (45.7%), and the lowest percentage in Sector 4 (0%), Sector 3 (13.3%), and Sector 5 (13%). Although sectors 1 and 2 had a higher percentage in the buccal-impacted group, sectors 3 and 5 were found to have a higher percentage in the palatal-impacted group (c^2^ = 35.32, *p* < 0.01) (Table 2).

The contact relationship was highest at the crown level (32.6%) and lowest at the apical level (17.4%) (c^2^ = 48.80, *p* < 0.001). The overall prevalence of root resorption was 56.5%, as presented in Table 2. The severity of resorption between canine and lateral incisors was observed to be highest in grade 1 (32.6%) and lowest in grade 3 (2.2%). A statistically significant positive association (c^2^ = 33.84, *p* < 0.001) was found between the groups (Table 2).

The mean values of the angles of U3 Ang showed statistically significant higher values in the BC and PC groups compared to the CC group (*p* < 0.001). Similarly, U3/U2 values showed significant variations. The mean values of the angulation of U4 were significantly higher in the BC group than in the CC group (*p* < 0.05) (Table 3).

In the mean values of U3 L, U2 L, U3 RL, and U2 RL, significant differences were found between the PC and CC groups. The mean values of U2 RL were statistically significantly lower in the BC group (*p* < 0.05) and PC group (*p* < 0.001) than in the CC group. The mean values of U3L were found to be statistically significantly lower in the PC groups compared to the BC group (*p* < 0.05).

There were differences in the mean values of U3 VD and U3 HD between the BC and CC groups, and between the PC group and the CC group (*p* < 0.001). The mean values of U3 HD were found to be lower in the PC group compared to the BC group (*p* < 0.001).

The mean values of U3 Inc were found to be statistically significantly higher in the BC (*p* < 0.01) and PC groups (*p* < 0.001), compared to the CC groups. The mean values of U3 RD and U2 RD were found to be lower in the BC (*p* < 0.05) and PC groups (*p* < 0.001) compared to the CC group. However, similar values were observed for the mean U4 RD angle values between the groups.

## 4. Discussion

Several factors should be considered during the planning of orthodontic treatment for impacted canines. These factors include the angulation of the maxillary impacted canine, inclination, horizontal and vertical location, age, malocclusion, surrounding tissue pathologies, and the amount of resorption in the root of the adjacent tooth [17,19,25,26,27,28,29]. Although, the frequency of impacted maxillary canines was found to be twice as high in females [30,31]. In this study, it was also found to be higher in females than in males.

CBCT provides the most useful information about the location and angulation of the maxillary impacted canine tooth, as well as the detection of resorption and pathology in the surrounding tissues. It is a very useful tool for accurate treatment planning [32]. Therefore, we preferred to use CBCT images in this study. In addition, while planning this study, we selected unilaterally impacted canine cases and canines from the non-impacted side of these cases as the control group to achieve more accurate and reliable results and to eliminate the differences between individuals. In addition, the participants of this study were individuals between the ages of 10–16. Radiographic studies have shown that eruption problems of maxillary canines before the age of 10 years are not suitable for assessing treatment prognosis [26].

Warford et al. conducted a study on panoramic radiographs to determine whether the combination of impacted tooth angulation values and sector location could predict greater impaction risk than sector location alone. Consequently, the location of the unerupted canine was found to be the most important contributor to impaction. There is a greater than 0.87 possibility of impaction when the canine overlaps the mid-long axis of the lateral incisor [27].

In this study, while sectors 1 and 2 had a higher percentage in the buccal-impacted group, sectors 3 and 5 had a higher percentage in the palatal-impacted group. The impacted canines in the palatal impacted group were located mesially closer to the midline. In addition, the vertical distance of the impacted canine was found to be greater in the palatally impacted group, while the midline distance was found to be greater in the buccally impacted group. At the same time, a study has shown that if impacted canines are positioned more mesially rotated and closer to the midline, they are more difficult to treat [26,30,33].

If the angle of the impacted canine with the midline is more than 31 degrees, the possibility of eruption of the impacted canine is considerably reduced [34]. At the same time, the increase in lateral incisor angulation with the impacted canine is also thought to be related to the impacted canine [29]. Ericson et al. stated that if the angulation of the impacted canine with the midline exceeds 25 degrees, and with the lateral incisor exceeds 28 degrees, the possibility of resorption increases by 50% [35].

While canines with inappropriate angulation continue to erupt, they produce abnormal pressures on the adjacent teeth and cause resorption. According to these results, it would be useful to assess the angulation of the impacted canine and its relationship to the lateral incisor in the mixed dentition period and to take precautions in the early period before resorption. In a study emphasizing the importance of the early detection of canine impaction, it was recommended that treatment should be performed before the apical third of the canine root is formed [36].

In this study, canine angulation was higher on the impacted side compared to the non-impacted side, while lateral angulation was lower. In the palatal-impacted canine group of this study, the canine angulation value (34.69°) was higher than the buccal-impacted canine group (26.23). In terms of the resorption rate and canine angulation, the severe resorption rate was found to be higher in the palatal group (grades 2 and 3). Long-term follow-up of teeth with root resorption in the adjacent region due to ectopically erupted canines showed no problems. In most of these cases, either the resorption regresses spontaneously or after surgery. Resorption decreases when the ectopically erupted canine is removed, or its position is changed by orthodontic treatment [37].

Al-Tawachi et al. reported that the root lengths of the lateral incisors in the palatal-impacted canine group were shorter than in the buccal-impacted and control groups, while the mesiodistal and buccopalatal widths were similar in all groups. In addition, they said that the lateral incisors were inclined distally and palatally [21]. Koral et al. reported that the width of the BL and MD of the lateral incisor, the length of the lateral incisor, the length of the root, the angulation of the midline of the lateral incisor, and the angle of the lateral incisor with the canine tooth are among the strong predictor factors of maxillary canine impaction [38].

BL and MD width of the lateral incisors in the PC group were smaller than in the other groups. However, the U2 RL and U2 L were smaller by 0.82–2.31 mm compared to those in the CC group and smaller in the PC group compared to the BC group, and the study’s hypothesis was rejected. These findings are also consistent with previous research indicating that the length of the lateral incisors is shorter on the impacted side [21,28,33,38,39,40].

The limitations of the present study include its retrospective design at a single center. In this study, we could only include CBCT images that fully captured the maxillary region. The findings of this study may be helpful in facilitating the diagnosis of maxillary impacted canines, preventing problems, and planning effective treatment. More research is needed to evaluate the angular and linear measurements on CBCT images in a wider population. One of the limitations of this study is that confounders were not included in the analysis. Adjusting for confounders in future studies will make the conclusions more robust.

As technology develops, future studies may apply artificial intelligence to various imaging modalities to provide information on predicting the maxillary canine impaction and treatment prognosis.

The lateral root length adjacent to the impacted canine was shorter, and the root dilaceration angle was smaller [28,39]. Similarly, this study found that adjacent lateral incisor roots were shorter, and that dilaceration of canine/lateral root in the impaction group was increased compared to the control group. These problems during eruption also cause root development problems.

As described in the eruption guidance theory, the roots of the lateral incisors guide the erupting permanent canines. In this study, it is clearly seen that the lateral incisor length and lateral incisor root length were found to be short in the BC and PC groups, which supports this theory. In other words, it should be considered that lateral incisors with shorter root lengths may be a potential cause for the maxillary canine impaction.

## 5. Conclusions

The maxillary impacted canines are more vertically positioned, mesiopalatally rotated, and in contact with adjacent teeth at the crown level.

Palatally impacted canines are horizontally closer to the midline and vertically higher from occlusion. Buccally impacted canines are positioned away from the midline and vertically lower. In addition, canine angulations are higher in the palatal group, making them more difficult to treat and increasing the degree of root resorption. Considering the possibility of resorption in these teeth in the clinic, orthodontic treatment that does not increase resorption with light forces should be applied.

The widths of the lateral incisor crowns were smaller in the palatally impacted canine group, although there was no statistically significant difference. These findings are one of the potential predictor factors indicating that the canine may remain impacted according to the guidance theory.

The fact that the U2 RL and U2 L values in the PC were lower than those in the BC suggests that the reduced dimensions of the maxillary lateral incisors and shorter roots may be a potential factor in the impaction of the maxillary canine.

## Figures and Tables

**Table 1:** Some demographic data of the groups and participants.

		Buccal (BC) n = 15	Palatal (PC) n = 16	Control (CC) n = 15	Total n = 46	χ^2^	df	*p*
Age	Mean ± SD	14.53 ± 2.55	14.87 ± 2.39	14.33 ± 2.02	14.58 ± 2.29	-	2	0.808 ^b^
Gender	n (%)							
	Female	8 (53.3%)	7 (43.8%)	9 (60%)	24 (52.2%)	0.831 ^a^	2	0.660
	Male	7 (46.7%)	9 (56.3%)	6 (40%)	22 (47.8%)			
Impaction side n (%)								
	Left side	9 (60%)	8 (50%)	9 (60%)	23 (50%)	1.200 ^a^	2	0.549
	Right side	6 (40%)	8 (50%)	6 (40%)	23 (50%)			

*p* levels were calculated by Pearson chi-square ^a^ test and one-way ANOVA ^b^.

**Table 2:** Percentages of location, rotation, sectors, contact point, and resorptions of impacted canines in the groups.

	Buccal (BC)	Palatal (PC)	Control (CC)	Total			
	n:15	n:16	n:15	n:46	χ^2^	Effect Size (V)	*p*
**Location**							
Vertical	15 (100%)	12 (75%)	15 (100%)	42 (91.3%)	6.02 ^c^	0.42	0.028
Horizontal	0 (0%)	4 (25%)	0 (0%)	4 (8.7%)			
**Rotation**							
No Rotation	0 (0%)	0 (0%)	15 (100%)	15 (32.6%)	48.08 ^c^	**0.71**	**<0.001**
Mesiobuccal	0 (0%)	0 (0%)	0 (0%)	0 (0%)			
Distobuccal	2 (13.3%)	4 (25%)	0 (0%)	6 (13%)			
Mesiopalatal	13 (86.7%)	12 (75%)	0 (0%)	25(54.3%)			
Distopalatal	0 (0%)	0 (0%)	0 (0%)	0 (0%)			
**Sectors**							
1	4 (26.7%)	2 (12.5%)	15 (100%)	21 (45.7%)	35.32 ^c^	**0.65**	**<0.001**
2	9 (60%)	4 (25%)	0 (0%)	13 (28.3%)			
3	2 (13.3%)	4 (25%)	0 (0%)	6 (13%)			
4	0 (0%)	0 (0%)	0 (0%)	0 (0%)			
5	0 (0%)	6 (37.5%)	0 (0%)	6 (13%)			
**Contact relation**							
Crown	0 (0%)	0 (0%)	15 (100%)	15 (32.6%)	48.80 ^c^	**0.74**	**<0.001**
Cervical	8 (53.3%)	4 (25%)	0 (0%)	12 (26.1%)			
Middle	5 (33.3%)	6 (37.5%)	0 (0%)	11 (23.9%)			
Apical	2 (13.3%)	6 (37.5%)	0 (0%)	8 (17.4%)			
**Resorption**							
Grade 0	4 (26.7%)	1 (6.3%)	15 (100%)	20 (43.5%)	33.84 ^c^	**0.60**	**<0.001**
Grade 1	8 (53.3%)	7 (43.8%)	0 (0%)	15 (32.6%)			
Grade 2	3 (20%)	7 (43.8%)	0 (0%)	10 (21.7%)			
Grade 3	0 (0%)	1 (6.3%)	0 (0%)	1 (2.2%)			

n = sample size; % = percentage of simple size. (V): Cramer’s V Statistical significance as determined by chi-square test (Fisher–Freeman–Halton Exact test ^c^), (*p* < 0.05).

**Table 3:** The mean values and standard deviation of measurements and comparison between groups.

	Buccal (BC)	Palatal (PC)	Control (CC)			Post Hoc		
	Mean ± SD	Mean ± SD	Mean ± SD	*p*	η^2^	BC-PC	BC-CC	PC-CC
**U3 Ang**	26.23 ± 13.46	34.69 ± 12.16	11.90 ± 3.07	**<0.001**	0.366	0.118	**0.020**	**<0.001**
**U2 Ang**	8.87 ± 6.67	10.84 ± 6.17	13.75 ± 2.63	0.178	0.091	0.787	0.063	0.318
**U4 Ang**	10.57 ± 6.31	6.10 ± 2.80	5.00 ± 2.93	**0.009**	0.232	0.061	**0.027**	0.781
**U3/U2**	27.94 ± 12.01	39.84 ± 16.20	4.12 ± 1.36	**<0.001**	0.533	0.080	**<0.001**	**<0.001**
**U3 BL**	8.40 ± 0.82	8.23 ± 0.77	8.29 ± 0.22	0.786	0.013	0.901	0.947	0.985
**U2 BL**	6.66 ± 0.66	6.47 ± 0.69	6.86 ± 0.47	0.382	0.052	0.708	0.757	0.361
**U3 MD**	8.26 ± 0.48	8.33 ± 0.72	8.19 ± 0.21	0.851	0.009	0.986	0.957	0.874
**U2 MD**	6.82 ± 0.78	6.57 ± 0.71	6.87 ± 0.45	0.491	0.039	0.580	0.983	0.574
**U3 RL**	13.37 ± 1.34	13.29 ± 1.18	14.59 ± 0.98	**0.043**	0.161	0.980	0.070	**0.047**
**U2 RL**	10.92 ± 0.95	10.33 ± 1.34	12.16 ± 0.45	**0.001**	0.305	0.284	**0.030**	**0.001**
**U3 L**	23.84 ± 1.80	21.95 ± 1.89	23.87 ± 1.68	**0.011**	0.223	0.017	0.999	**0.050**
**U2 L**	20.50 ± 1.55	19.01 ± 2.68	21.32 ± 1.60	**0.033**	0.173	0.133	0.651	**0.041**
**U3 VD**	7.64 ± 3.97	9.23 ± 3.00	00.00 ± 0.00	**<0.001**	0.572	0.528	**<0.001**	**<0.001**
**U3 HD**	14.79 ± 2.37	6.10 ± 4.12	16.75 ± 1.12	**<0.001**	0.713	**<0.001**	**0.041**	**<0.001**
**U3 Inc**	32.11 ± 14.68	35.45 ± 14.38	18.23 ± 3.54	**0.015**	0.208	0.895	**0.009**	**0.001**
**U2 Inc**	17.94 ± 9.33	19.49 ± 9.21	24.29 ± 4.85	0.246	0.075	0.870	0.223	0.409
**U3 RD**	150.35 ± 15.74	148.87 ± 13.92	172.21 ± 6.63	**0.001**	0.329	0.951	**0.002**	**0.001**
**U2 RD**	161.76 ± 11.36	158.84 ± 10.06	171.54 ± 4.56	**0.017**	0.201	0.839	**0.025**	**0.001**
**U4 RD**	157.35 ± 8.86	160.61 ± 10.02	165.94 ± 4.93	0.096	0.122	0.561	0.079	0.351

One-way ANOVA test for those with normal distribution, *p* < 0.05 post hoc for homogeneous distribution: Tukey test and Tamhane’s T2 test for those not homogeneously distributed, *p* < 0.05 Kruskal–Wallis test for those who do not have a normal distribution. η^2^: (eta^2^, effect size), Ang: angulation, BL: buccolingual, MD: mesiodistal, RL: root length, L: length, VD: vertical distance, HD: horizontal distance, Inc: inclination, and RD: root dilaceration.

## Data Availability

The data presented in this study are available upon request from the corresponding authors.
